# The Auscultation of a Carbon Dioxide Embolization Event during Endoscopic Vein Harvest

**DOI:** 10.1155/2016/6947679

**Published:** 2016-05-31

**Authors:** Erik Strauss, Bradley Taylor, Michael Mazzeffi, Kenichi Tanaka, Patrick Odonkor

**Affiliations:** ^1^Department of Anesthesiology, University of Maryland School of Medicine, Baltimore, MD 21201, USA; ^2^Department of Surgery, University of Maryland School of Medicine, Baltimore, MD 21201, USA

## Abstract

Endoscopic vein harvest in preparation for coronary artery bypass surgery has become a preferred method of procuring saphenous vein. Several case reports have documented carbon dioxide (CO_2_) embolization with this procedure as well as CO_2_ embolization during other laparoscopic surgeries (Markar et al., 2010). Although uncommon, the potential for CO_2_ entrainment through an open vein or through absorption by vascular structures exists and should be recognized (Lin et al., 2003). We report a case of CO_2_ embolization during EVH for a 77-year-old patient who underwent CABG that was identified early by the cardiac surgeon through the indirect auscultation of a mill-wheel murmur after the pericardium was opened. This may be the first reported case of a murmur related to air emboli identified without the use of a precordial Doppler probe or a stethoscope. This diagnosis was further supported by TEE before systemic hypotension or cardiovascular collapse occurred.

## 1. Introduction

Endoscopic vein harvest (EVH) has a number of benefits compared to the open technique including less postoperative pain, wound infections, and hematomas [1] but potential complications of EVH should not be overlooked. One such complication reported in multiple case reports is carbon dioxide embolization leading to hemodynamic collapse. The reported incidence of moderate and severe CO_2_ embolization during EVH is 3.5% and 0.5%, respectively, while mild CO_2_ embolization has been reported at an incidence of 13.1%. Evidence of small CO_2_ bubbles in the inferior vena cava can be seen often using transesophageal echocardiography (TEE) even during straightforward or unremarkable EVH which shows no effects on the patient's hemodynamics.

## 2. Case Report

A 77-year-old man with a history of HTN and recently diagnosed multivessel CAD was brought to the operating room for a four-vessel CABG. The patient received general anesthesia with an endotracheal tube, a right radial artery catheter, right internal jugular introducer, and pulmonary artery catheter. The saphenous vein harvest using the VASOVIEW HEMOPRO 2 Endoscopic Vessel Harvesting System (Maquet) started concurrently with sternal incision. The EVH procedure was challenging and insufflation pressure initially 12 mmHg was increased to 14 mmHg while an accumulating amount of blood could be seen in the saphenous vein tunnel. During left internal mammary artery (LIMA) takedown after an uncomplicated sternotomy there was an increase in end-tidal CO_2_ from 32 mmHg to 44 mmHg and an increase in PA pressure from 24/14 to 34/20 mmHg during EVH. These changes were assumed to be caused by relative hypoventilation as the tidal volumes were decreased to 300 mL with a respiratory rate of 12/min to improve surgical visualization during LIMA takedown. Less than five minutes after opening the pericardium with the EVH continuing the attending surgeon heard a murmur coming from the surgical field and asked for complete silence in the operating room. The surgical assistants and the attending anesthesiologist heard a subtle sound similar to that of a quiet washing machine coming from the heart. The surgeon suggested that the new murmur could be due to CO_2_ embolization and asked that endoscopic harvest be immediately stopped. TEE imaging of the right ventricle and pulmonary artery showed a large amount of bubbles most likely CO_2_ emboli. Further images in the bicaval view showed bubbles coming from the IVC. TEE images were saved less than sixty seconds after insufflation was stopped which is why the images show only a fleeting glimpse of the gas emboli. ABG drawn one minute before the murmur was heard showed pH 7.27 with PaCO_2_ 67 mmHg consistent with systemic CO_2_ absorption and helped to confirm the diagnosis of CO_2_ embolization. The murmur had stopped by the time the first TEE images were taken and the patient's systemic blood pressure and heart rate remained within physiologic limits.

## 3. Discussion

In retrospect the signs of CO_2_ embolization were present minutes before the murmur was heard. The increase in PA pressure and end-tidal CO_2_ were present minutes prior to the diagnosis and the difficulty with the saphenous vein harvest and increased insufflation pressure should have raised awareness that significant CO_2_ embolization could occur. Almost certainly the gas emboli would have been seen on TEE imaging during this time. Although it is not routine practice at our institution to keep the TEE imaging on during EVH to monitor for CO_2_ emboli, it would have been useful in this case. Since CO_2_ gas is easily absorbed in blood and expired from the lungs, there is a larger margin of safety using this gas compared to room air and in most cases a larger amount of CO_2_ emboli must enter the circulatory system before hemodynamic collapse occurs. In cases of massive CO_2_ embolization during EVH the CO_2_ entrainment into the circulatory system is probably occurring over the course of a few minutes time as it was in this case. Awareness of the factors that increase the likelihood of CO_2_ emboli can lead to an earlier diagnosis as it is probably unnecessary and unrealistic to expect that practitioners continuously and routinely use TEE during EVH to monitor for CO_2_ emboli but it should be used as monitor when the suspicion is high.

Difficulty during EVH where it is more likely that open or unclipped branches of the vein are exposed to CO_2_ insufflation pressure correlates with increased CO_2_ embolization events [2]. There is a higher incidence of CO_2_ emboli as demonstrated in a porcine model when the CVP is lower than the insufflation [3]. The Maquet company recommends a CO_2_ gas flow of 3–5 L/min and a tunnel pressure of 10–12 mmHg for the VASOVIEW HEMOPRO 2 during saphenous vein harvest. A prospective randomized study showed that a CO_2_ insufflation pressure of 12 mmHg had a significantly lower incidence (6.5%) of CO_2_ emboli seen in the IVC on TEE than a CO_2_ insufflation pressure of 15 mmHg (13.3%) [4].

In this case it was the classic “mill-wheel murmur” caused by gas accumulating in the cardiac chambers and splashing at the air fluid interface throughout the cardiac cycle that led to the first suspicion of CO_2_ embolization. A previous animal model showed that this mechanism generates the murmur [5]. A precursory literature search showed no previous descriptions of the mill-wheel murmur heard without the aid of a precordial Doppler probe or stethoscope leading to this diagnosis. Perhaps the open pericardium made it easier for the murmur to be heard but most likely there was a large amount of CO_2_ gas present in the right ventricle enough to classify it as submassive CO_2_ embolization. Fortunately the diagnosis was made before hemodynamic collapse occurred as we literally heard it coming.

## 4. Conclusion

CO_2_ embolization during EVH is common but only in small amounts that have no hemodynamic consequences. TEE should be used to monitor for large CO_2_ embolization events if ETCO_2_ levels or PA pressures abruptly increase during EVH, CO_2_ insufflation pressures > 12 mmHg are used, or saphenous vein harvesting appears difficult or complicated. If the sternum and pericardium are opened, a mill-wheel murmur can be heard due to CO_2_ gas accumulation in the right ventricle as a final warning sign before hemodynamic collapse.

## Supplementary Material

Transesophageal echocardiography showing a modified view of the right ventricular outflow tract. Gas emboli can be seen in the right ventricular outflow tract as well as a pulmonary artery catheter. Image was acquired less than one minute after endoscopic vein harvest had stopped. Images not saved showed significantly more gas in the RVOT, which very quickly dissipated once insufflation had stopped.
